# Phenolic Compounds from *Merremia umbellata* subsp. *orientalis* and Their Allelopathic Effects on Arabidopsis Seed Germination

**DOI:** 10.3390/molecules15118241

**Published:** 2010-11-12

**Authors:** Jian Yan, Hai-Hong Bi, Yong-Zhu Liu, Mei Zhang, Zhong-Yu Zhou, Jian-Wen Tan

**Affiliations:** 1 Key Laboratory of Plant Resources Conservation and Sustainable Utilization, South China Botanical Garden, Chinese Academy of Sciences, Guangzhou 510650, China; E-Mails: yanjian@scbg.ac.cn (J.Y.); bihaihong@scau.edu.cn (H.-H.B); amei1227@126.com (M.Z.); zhouzhongyu@scbg.ac.cn (Z.-Y.Z.); 2 National Engineering Research Center of Plant Space Breeding, Guangzhou 510642, China; E-Mail: lively@scau.edu.cn; 3 Graduate School of the Chinese Academy of Sciences, Beijing 100039, China

**Keywords:** Convolvulaceae, *Merremia umbellata*, phenolic compounds, *Arabidopsis*, Allelopathic effect, salicylic acid

## Abstract

A bioassay-directed phytochemical study was carried out to investigate potential allelochemicals of the invasive plant *Merremia umbellata* subsp. *orientalis* (Hall. f.). Eight phenolic compounds, including a salicylic acid (SA)-derived new natural product, SA 2-*O*-β-D-(3′,6′-dicaffeoyl)-glucopyranoside (**1**), and seven known ones **2**-**8** were isolated and identified from two bioactive sub-fractions of the acetone extract of this plant. The structure of new compound **1** was established by spectral and chemical methods. The potential allelopathic effects of these compounds at 0.5 and 1.0 mM concentrations on the germination of *Arabidopsis* seeds were tested. Results showed that **2** remarkably inhibited seed germination at concentrations as low as 0.5 mM. Compound **3** only moderately inhibited seed germination at 0.5 mM, but displayed strong inhibitory bioactivity at 1.0 mM concentration. Compounds **4** and **5** showed only slight inhibitory bioactivity at 1.0 mM, while the other compounds showed no obvious inhibitory effects.

## 1. Introduction

**Figure 1:**
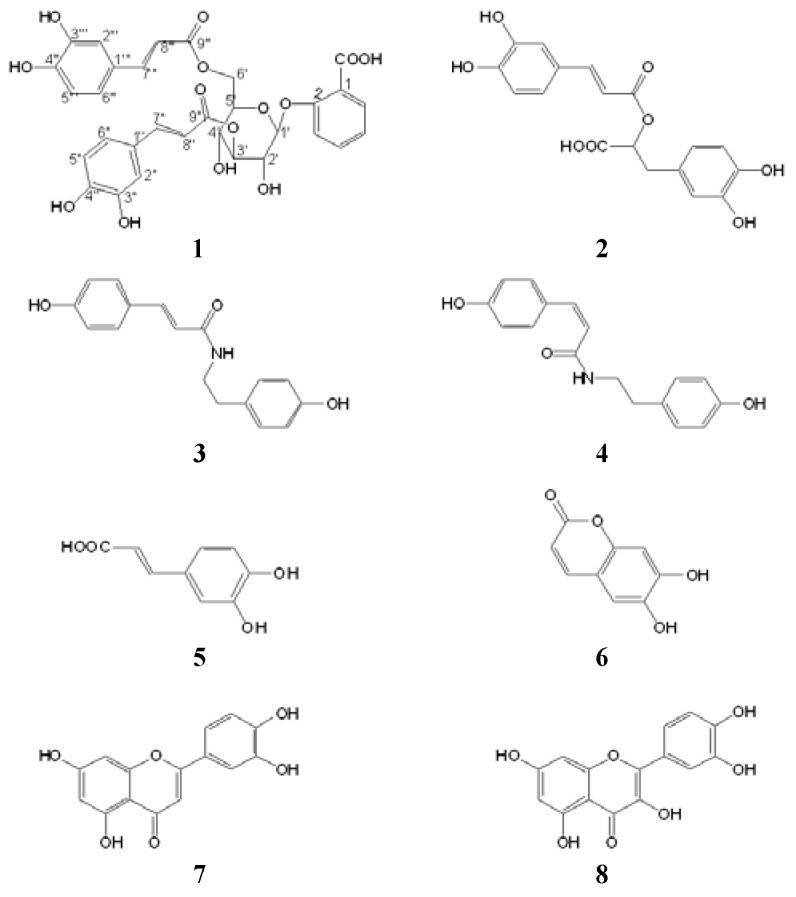
Phenolic compounds isolated from *M. umbellata* subsp. *orientalis.*

*M. umbellata* (Linn.) subsp. *orientalis* (Hall. f.) belonging to the family Convolvulaceae is a typical liana plant found in many tropical and subtropical Asian countries like India, Vietnam and Laos, and this plant is also recorded in Australia, eastern Africa and some Pacific Ocean islands [1]. In China, this plant was previously mainly distributed in Guangxi, Yunnan and Hainan provinces, while recently it was also found in some parts of Guangdong Province as an introduced harmful invasive plant [2]. Just like some other well-known invasive plants such as *Centaurea maculosa* in North America [3] and *Eupatorium adenophorum* in Asia [4], it could be speculated that the generation of potential allelochemicals would possibly be an important factor for this plant to achieve its invasive success. However, though diverse resin glycosides [5,6,7,8,9,10], phenolic compounds [11,12,13,14], furanosesquiterpenoids [15], pyrrolidine and tropane alkaloids [16] have been identified from some *Merremia* genus plants, no particular chemical constituents of this plant or their potential allelopathic activities have been reported so far. As one part of our study on invasion mechanisms of invasive plants in China, we carried out a bioassay-directed phytochemical investigation on potential allelochemicals of *M. umbellata* subsp. *orientalis*, which led to the isolation and identification of eight phenolic compounds, including a new salicylic acid (SA)-derived natural product, SA 2-O-β-D-(3′,6′-dicaffeoyl)-glucopyranoside (**1**), and seven known ones (**2**-**8**, see Figure 1) from two bioactive sub-fractions (Fr.2 and Fr.5) of an acetone extract of this plant. 

Here we report the isolation and structure elucidation of these compounds and their potential allelopathic effects tested on the germination of *Arabidopsis* seeds.

## 2. Results and Discussion

**Figure 2:**
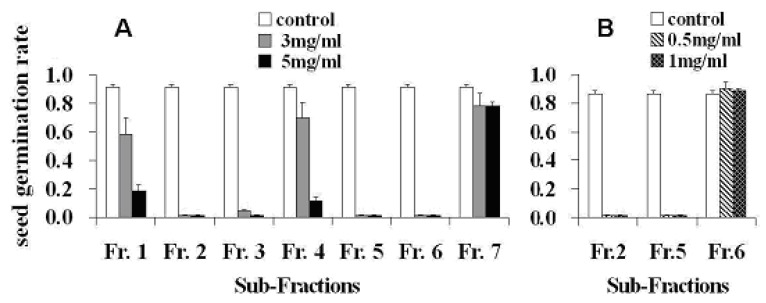
Arabidopsis seed germination rates recorded after three days in Hoagland medium containing sub-fractions (Fr.1-Fr.7) of the acetone extract of the plant fresh tissues at the indicated concentrations or nothing (A: bioactive assay for all seven sub-fractions at 3.0 and 5.0 mg/mL concentrations; B: further bioactive assay for Fr.2, Fr.5 and Fr.6 at 0.5 and 1.0 mg/mL concentrations; control: filter paper treated with 3 mL of pure acetone). Values are means ± s.d. from three independent experiments (30 seeds per treatments). Seeds germination rate ＝ (number of germinated seeds/30) × 100%.

The acetone extract of whole plant fresh tissues of *M. umbellata* subsp. *orientalis* was partitioned with EtOAc and 10% ethanol in H_2_O, and the obtained EtOAc extract was subsequently subjected to silica gel column chromatography, eluted with a CHCl_3_/MeOH system, to afford seven sub-fractions (Fr.1-7). The allelopathic effects of the seven sub-fractions on the germination of *Arabidopsis* seeds were tested by using a literature method [4], as described in the Experimental, and the results showed that sub-fractions Fr.2 and Fr.5 were capable of remarkably inhibiting the germination of *Arabidopsis* seeds at concentrations as low as 0.5 mg/mL (Figure 2). This suggested that potential allelochemicals of the plant in the acetone extract would have mainly been partitioned and concentrated into Fr.2 and Fr.5, therefore these sub-fractions were further phytochemically analyzed in the search for potential allelochemicals, which eventually led to the isolation of compounds **1**, **2** and **3** from Fr.5 and **4**, **5**, **6**, **7** and **8** from Fr.2.

Compound **1** was obtained as a white, amorphous powder. Its HR-ESI-MS exhibited a molecular ion peak at *m/z* 623.1422 [M-H]^-^ corresponding to a molecular formula of C_31_H_28_O_14_ (calcd. 623.1400). IR absorptions at 3,374 cm^-1^ (br.) and 1,695 cm^-1^ (br.), implied the existence of hydroxyl and conjugated carbonyl groups. In its ^13^C-NMR and DEPT spectra, the thirty-one carbon signals of the molecule (11×C, 19×CH, 1×CH_2_, three carbonyl group C-atoms and twenty-two *sp^2^ C-atoms) could all be assigned (see Table 1). The presence of a* D-glucopyranose moiety in the molecule was suggested by the presence of carbon signals at *δ*_C_ 103.9 (CH), 73.3 (CH), 78.1(CH), 70.0 (CH), 75.8 (CH) and 64.3 (CH_2_), confirm by the presence of signals at *δ*_H_ 5.04 (1H, H-1′), 3.78 (1H, H-2′), 5.19 (1H, H-3′), 3.68 (1H, H-4′), 3.87 (1H, H-5′), 4.56 (1H, Ha-6′) and 4.44 (1H, Hb-6′) in its ^1^H-NMR spectrum. 

**Figure 3:**
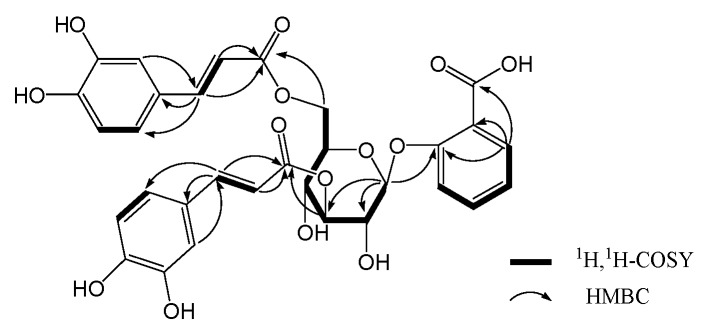
Key HMBC and COSY correlations of compound **1**.

Coupled with ^1^H-^1^H COSY and HSQC spectral analysis, aromatic proton signals at *δ*_H_ 7.81 (1H, H-3), 7.42 (1H, H-4), 7.10 (1H, H-5) and 7.34 (1H, H-6) belonging to a salicylic acid moiety, and aromatic and olefinic proton signals of two caffeic acid moieties at *δ*_H_ 7.07 (1H, H-2″), 6.80 (1H, H-5″), 6.79 (1H, H-6″), 7.06 (1H, H-2‴), 6.78 (1H, H-5‴), 6.69 (1H, H-6‴), and *δ*_H_ 7.62 (1H, H-7″), 6.36 (1H, H-8″), 7.59 (1H, H-7‴), 6.31 (1H, H-8‴), were all observed (see Figure 3). The presented coupling constants between H-7″(7‴) and H-8″(8‴) olefeinic protons (*J*_7″,8″_ = 16.2 Hz, *J*_7‴,8‴_ = 15.6 Hz) suggested that the two double bonds in the two caffeic acid moieties, respectively, were all *E* geometry. In the HMBC spectrum, the observation of ^1^H-^13^C long-range correlations of H-7″(7‴) with C-2″(2‴) and C-6″(6‴), H-8″(8‴) with C-1″(1‴) and C-9″(9‴) indicated the direct linkage of C-1″(1‴) with C-7″(7‴), and C-8″(8‴) with C-9″(9‴). The bonding of the 7-COOH group with C-1 was supported by the observation of a significant HMBC correlation of H-6 (*δ*_H_ 7.34) with C-7 (*δ*_C_ 169.7). The 2-O-*β*-D-glucopyranoside linkage was assigned based on the HMBC correlation of H-1′ with C-2 and the coupling constant of the anomeric proton (H-1′, *δ*_H_ 5.04, *d*, *J* = 7.8 Hz) shown in its ^1^H-NMR spectrum. The ester bond linkage of two caffeic acid units with the sugar moiety at C-3′ and C-6′ positions were revealed by the observation of significant HMBC correlations of H-3′ with C-9″ and H-6′ with C-9‴. Those spectral data led us to establish the structure of **1** as SA 2-*O*-β-D-(3′,6′-dicaffeoyl)-glucopyranoside.

In order further to confirm the configuration of the glucose moiety in the new structure, the permethylated product **1(X)** prepared from the sugar unit released by hydrolyzation from **1** was compared by GC-MS analysis with permethylated products **D(**+**)** and **L(-)** prepared from authentic D- and L-glucose, respectively. Results showed that **1(X)** (with retention time of 3.39 min) was consistent with **D(**+**)** (with retention time of 3.38 min), but obviously different from **L(-)** (with a retention time of 3.67 min), indicating that the sugar moiety in **1** was a D-glucose unit, which further confirmed our deduction about the structure of **1** as SA 2-*O*-β-D-(3′,6′-dicaffeoyl)-glucopyranoside.

The seven known phenolic compounds were identified as rosmarinic acid (**2**) [17,18], paprazine (**3**) [19], *N*-*P*-*cis*-coumaroyltyramine (**4**) [20], caffeic acid (**5**) [18,21], esculetin (**6**) [22], quercetin (**7**) [22,23], and luteolin (**8**) [24], respectively, by comparison of their spectral data with those in the literature. All of these compounds were isolated from *M. umbellata* subsp. *orientalis* for the first time.

**Figure 4:**
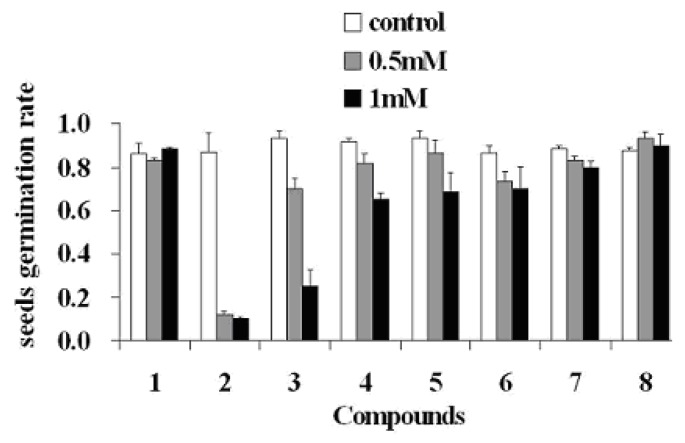
Arabidopsis seed germination rates recorded after three days in Hoagland medium containing one of the isolated compounds **1**-**8** at the indicated concentrations or nothing (control: filter paper treated with 3 mL of pure acetone). Bars indicate maximal deviations from mean values derived from three independent measurements. Seeds germination rate ＝ (number of germinated seeds/30) × 100%.

Interference of invasive plants with the growth and establishment of native plants via allelopathy has been indicated in a number of recent studies as one of the most important factors for invasive plants to achieve their invasion success. So far, structurally diverse allelochemicals have already been discovered in several invasive species, such as catechin from *Centaurea maculosa* [3] and various sesquiterpenoids from *Eupatorium adenophorum* [4], *etc*. The allelopathic effects of the phenolic compounds obtained in this study were tested at 0.5 mM and 1.0 mM concentrations, respectively, on the germination of *Arabidopsis* seeds, using a literature method [4] as described in the Experimental section, in which the seeds’ germination rates three days post-innoculation were recorded. Results showed that **2** remarkably inhibited *Arabidopsis* seed germination at both concentrations. Compound **3** significantly inhibited seed germination at 1.0 mM, but showed only moderate bioactivity at 0.5 mM concentration. Compounds **4** and **5** only slightly inhibited the seed germination at 1.0 mM concentration, and no inhibitory effects were exhibited by the other compounds at concentrations tested (Figure 4).

Phenolic compounds functioning as allelochemicals have already been addressed in several different invasive plant species [3,25,26], among which catechin is so far the most comprehensively investigated one in the literature [27]. Our study indicated that several phenolic compounds in *M. umbellata* subsp. *orientalis* were also biologically active and they could also be potential allelochemicals. Since more than one compound with allelopathic potential was discovered in this plant, it could be speculated that synergistic effects of those potential allelochemicals could exist for this plant to exert its potential allelopathic effects. Compounds **7** and **8** are two typical flavonoids, whose structures are close to that of the highly bioactive compound catechin, but no obvious allelopathic effects were exhibited by these two compounds in our experiments, which suggests that the C-2, C-3 chairal centres and the β-OH group at C-3 of catechin might be necessary for the phytotoxicity of flavonoids of this type.

The new compound **1** showed no biological activity in our bioactivity assay, which in itself is interesting since **1** is a salicylic acid (SA)-containing molecule and according to the literature, SA is an important signaling molecule that mediates plant defense against microbial pathogens [28,29]. Since SAG has been confirmed to be an important slow-release storage form of SA for regulation of SA-elicited defense reactions [30], it is reasonable to predict that **1** might be a new storage form of SA in higher plants, or at least in this plant, to regulate SA-elicited defense responses.

## 3. Experimental

### 3.1. General

Uncorrected melting points (m.p.) were obtained on a Sichuan micro-melting-point apparatus (XRC-1). Optical rotations were measured on a Horiba SEPA-300 spectropolarimeter. IR spectra (KBr) were recorded on a Bruker Tensor 27 spectrophotometer in cm^-1^. 1D and 2D ^1^H- and ^13^C-NMR spectra were recorded in CD_3_OD on a Bruker AVANCE-600 instrument with TMS as an internal standard. ESIMS and HRESIMS were recorded on a VG-Autospec-3000 spectrometer. For column chromatography (CC), silica gel (200-300 mesh, Qingdao Marine Chemical Inc., Peoples Republic of China), Lichroprep RP-18 gel (40-63 um, Merck, Darmstadt, Germany) and Sephadex LH-20 (Pharmacia Fine Chemical Co. Ltd.) were used. Fractions were monitored by TLC, and spots were visualized by heating the silica gel plates sprayed with 10% H_2_SO_4_ in ethanol.

### 3.2. Plant Material

Whole plant fresh *M. umbellata* subsp. *orientalis* (Hall. f.) materials were collected at Longdong forest park in Guangzhou, Guangdong Province, Peoples Republic of China, in February 2009, and identified by Prof. Fuwu Xing, South China Botanical Garden, Chinese Academy of Sciences (CAS). The voucher specimen (NO: 20090222) was deposited at the Laboratory of Phytochemistry of the South China Botanical Garden, CAS.

### 3.3. Extraction and Isolation

The freshly collected *M. umbellata* subsp. *orientalis* material (7.7 kg) was cut into pieces and exhaustively extracted for three times with 80% acetone (in H_2_O) at room temperature (3 × 30 L). The acetone extract was then evaporated to dryness under reduced pressure to remove most of the acetone and re-suspended in 10% methanol in H_2_O (2,500 mL). The re-suspended solution was then extracted with EtOAc (2,000 mL × 3) and the EtOAc extract was subsequently evaporated to dryness to give 48 g of oily EtOAc extract. This EtOAc extract (48 g) was further subjected to column chromatography over silica gel column (200-300 mesh, 1.2 kg) eluted with a gradient solvent system of CHCl_3_/MeOH (0-100% methanol) to afford seven sub-fractions Fr.1-7. As a result of the bioassay-guided analysis, Fr.2 and Fr.5 were mainly investigated. Fr.2 was fractionated into five sub-fractions by CC. Fr. 2.2 (5.8 g) was repeatedly subjected to CC (silica gel, CHCl_3_/MeOH 25:1 to 10:1) to give compounds **5** (1 g, yield:17.24%), **6** (23 mg, 0.40%), **7** (20 mg, 0.34%) and **8** (11 mg, 0.19%), and further purified by prep. HPLC to yield compound **4** (12 mg, 0.21%). Fr.5 (2.1 g) was also repeatedly subjected to CC eluting with CHCl_3_/CH_3_OH (40:1) to afford compounds **2** (16 mg, 0.76%), **3** (60 mg, 2.86%), and further purified by Sephadex LH-20 column eluting with MeOH to give a white powder of the new compound **1** (10 mg, 0.48%). SA 2-O-β-D-(3′,6′-dicaffeoyl)-glucopyranoside (**1**). white powder; m.p.: 168-170 ºC; [α]^20^_D_: +85.5 (*c* 0.08, CH_3_OH); UV λ_max_ (CH_3_OH) nm (logε): 217 (4.39), 329 (4.39); IR (KBr) ν_max_ 3374, 1695, 1602, 1517, 1488, 1450, 1355, 1278, 1160, 1112 cm^-1^; ^1^H-NMR (CD_3_OD) data, see Table **1**; ^13^C-NMR (CD_3_OD) data, see Table **2**; ESI-MS (negative) *m/z* 623, HR-ESI-MS (negative): 623.1422 ([M-H]ˉ, calcd. for C_31_H_27_O_14_ˉ, 623.1400).

### 3.4. Hydrolysis, Sugar Permethylation and GC-MS Analysis

Compound **1** (2 mg) was refluxed with a mixture of MeOH (1 mL) and 5% H_2_SO_4_ (1 mL) at 90 ºC for 2 h. The reaction mixture was diluted with H_2_O (2 mL) and extracted with EtOAc (1.5 mL). The aqueous layer was neutralized with 10% NaHCO_3_ (1.1 mL) and concentrated to give a residue, in which the sugar unit released from **1** was contained. The residue was further dissolved in anhydrous DMSO (1 mL), KOH (1 mg) and CH_3_I (1 mL) added for permethylation, and the mixture was allowed to react at room temperature for 1 h. Then the reaction was stopped by addition of H_2_O (2 mL) and the reaction mixture was then extracted with CHCl_3_ (3 × 1.5 mL). The combined organic layers were washed with H_2_O (3 mL), and evaporated to afford the permethylated sugar product **1(X)**. The permethylated products **D(**+**)** and **L(-)** of authentic sugars D-glucose and L-glucose (Sigma), respectively, were prepared with the same method as for **1(X)**. GC-MS analyses was carried out to compare **1(X)** with **D(**+**)** and **L(-)** by using a GCMS-QP2010 PLUS instrument, RXI^R^-5ms capillary column (30m, 0.25mm ID), Helium at constant rate of 40 cm/s, 5 uL injection valume, injector temperature at 290 ºC, temperature program as 50 ºC for 3 min, then 15 ºC/min to 180 ºC, hold for 10 min, then 15 ºC/min to 280 ºC, hold for 8 min, then 40 ºC/min to 300 ºC, hold for 20 min. Electron ionization mode was used at 70 eV. The mass range was 100–900 amu. The temperature of the ion source was 220 ºC. The retention times detected were 3.39 min for **1(X),** 3.38 min for **D(**+**)** and 3.67 min for **L(-)**. The consistency of the retention time of **1(X)** with that of **D(**+**)** confirmed our deduction that the sugar moiety in **1** had a D-configuration.

### 3.5. Seed Germination Bioassay

*Arabidopsis* *thaliana* was washed with ethanol (70% v/v) for 2 min and surface sterilized using sodium hypochlorite (0.5% v/v) for 2 min, followed by three washes with sterile distilled water. After surface sterilization, seeds were stored in a refrigerator at 4 ºC for three days before use. Three layers of filter paper were put in 6-cm-diameter glass Petri dishes, and the filter papers were impregnated with the compounds dissolved in methanol (2 mL). Concentrations of sub-fractions and compounds were as indicated in Figure 2 and Figure 4. To avoid toxic effects of the organic solvent, filter paper treated with acetone solution was placed in a fume cupboard for 1 h to allow complete solvent evaporation [31]. Subsequently, Hoagland solution (1 mL) was added to each piece of filter paper in each Petri dish [32]. Thirty *Arabidopsis* seeds were evenly placed on the moist filter paper in each Petri dish. Two controls (filter paper treated with 2 mL of methanol and filter paper without any treatment) were used. Each treatment had three duplicates. Seeds were allowed to germinate under 12 h light and 12 h dark at 25 ºC (day) and 20 ºC (night). The light intensity in the growth chamber was 100 μmol m^-2^ s^-1^. The number of germinated seeds was recorded after treatment for three days, at which time most seeds (≥ 80%) in the control Petri dishes were germinated. 

## 4. Conclusions

Eight phenolic compounds, including a new salicylic acid-derived natural product SA 2-*O*-β-D-(3′,6′-dicaffeoyl)-glucopyranoside, and seven known ones were isolated from the invasive plant *M. umbellata* subsp. *orientalis* (Hall. f.), and some of them showed inhibitory bioactivities on the germination of *Arabidopsis* seeds. Our study provides new data to support the idea that phenolic compounds could play a role as allelochemicals in helping invasive plants achieve their invasion success. Our research does not exclude the possibility that some other types of allelochemicals would also exist in this plant, and this possibility could be gradually clarified with the functional identification of more chemical constituents of this plant in the future.

## Figures and Tables

**Table 1:** ^1^H- and ^13^C-NMR spectral data of compound **1**.

No.	*δ* (C)	*δ* (H)	No.	*δ* (C)	*δ* (H)
1	123.0		1″	127.9	
2	158.4		2″	115.3	7.07 (1H, d, 2.4)
3	119.2	7.81 (1H, dd, 7.8, 1.2)	3″	146.9	
4	134.9	7.42 (1H, dt, 7.8, 1.2)	4″	149.7	
5	124.0	7.10 (1H, dt, 7.8, 1.2)	5″	116.6	6.80 (1H, d, 8.4)
6	132.5	7.34 (1H, dd, 7.8, 1.2)	6″	123.0	6.79 (1H, dd, 8.4, 2.4)
7	169.7		7″	147.3	7.62 (1H, d, 16.2)
1′	103.9	5.04 (1H, d, 7.8)	8″	115.2	6.36 (1H, d, 16.2)
2′	73.3	3.78 (1H, dd, 9.6, 7.8)	9″	168.9	
3′	78.1	5.19 (1H, dd, 9.6, 9.0)	1‴	127.7	
4′	70.0	3.68 (1H, dd, 9.6, 9.0)	2‴	115.2	7.06 (1H, d, 1.8)
5′	75.8	3.87 (1H, m)	3‴	146.8	
6′a	64.3	4.56 (1H, dd, 12.0, 1.8)	4‴	149.6	
6′b		4.44 (1H, dd, 12.0, 6.6)	5‴	116.5	6.78 (1H, d, 8.4)
			6‴	122.9	6.69 (1H, dd, 8.4, 1.8)
			7‴	147.2	7.59 (1H, d, 15.6)
			8‴	114.9	6.31 (1H, d, 15.6)
			9‴	168.9	

Data were measured at 600 MHz for ^1^H and 150 MHz for ^13^C in CD_3_OD, *δ* in ppm and *J* in Hz.
